# Metastasis-directed therapy for oligometastatic urological tumours: still no second-hand news

**DOI:** 10.3332/ecancer.2020.1036

**Published:** 2020-05-07

**Authors:** Charlien Berghen, Steven Joniau, Christof Vulsteke, Maarten Albersen, Gaëtan Devos, Kato Rans, Karin Haustermans, Gert De Meerleer

**Affiliations:** 1 Department of Radiation Oncology, Leuven University Hospital, Leuven, Belgium; 2 Department of Urology, Leuven University Hospital, Leuven, Belgium; 3 Department of Oncology, Ghent Maria Middelares Hospital, Ghent, Belgium; 4 Department of Molecular Imaging, Pathology, Radiotherapy and Oncology (MIPRO), Center for Oncological Research (CORE), University of Antwerp, Antwerp, Belgium

**Keywords:** oligometastases, urological tumours, prostate cancer, bladder cancer, renal cancer, radiotherapy, metastasectomy

## Abstract

For patients presenting with limited metastatic disease burden, known as the oligometastatic state of disease, a more aggressive treatment approach targeting the new or progressive metastatic lesions might improve patient outcome, with no or only limited toxicity to be expected from the treatment. This review provides an overview of the existing evidence and on-going trials on oligometastatic disease and metastasis-directed therapy in the field of renal, bladder and prostate cancer.

## Introduction

The perspective on metastatic disease has changed dramatically, due to the availability of novel imaging modalities as well as molecular diagnostics and further improvement of available treatments. From a dichotomy between localised and metastatic disease towards the concept of a sequence of events acquiring metastatic ability [1], limited cancer spread is now seen as a transitional state between locally confined disease and widespread metastases. This state is called the oligometastatic state of disease [2], defined as the presence of limited metastatic lesions up to three or five [3, 4]. There are three general definitions of oligometastatic disease, implicating different treatment approaches and therapeutic goals [5]. In upfront oligometastatic disease (synchronous metastasis), the disease has spread to limited sites and these metastases are present at diagnosis, i.e., before initiation of any treatment. In case of oligorecurrent metastatic disease (metachronous metastases), a limited number of metastases will develop some time after treatment with curative intent of the primary tumour. *Oligoprogression* is defined as the appearance and/or progression of a limited number of lesions in a metastatic patient, while all other lesions are being controlled by on-going systemic treatment.

**Figure 1.**
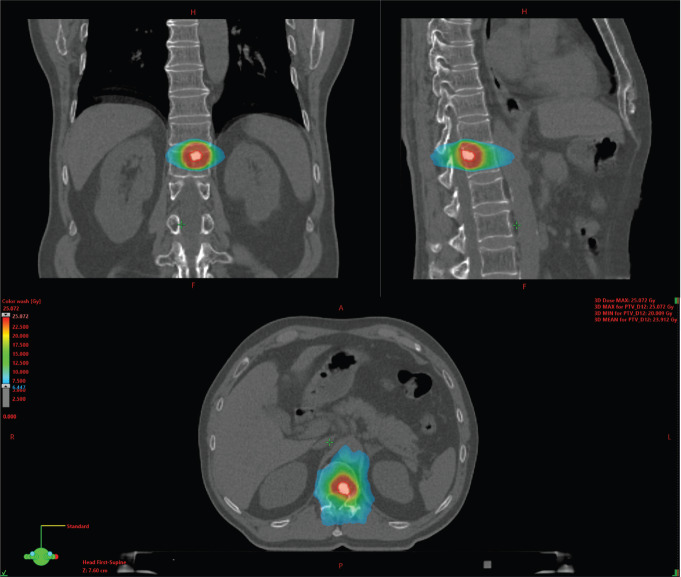
Example of a stereotactic body radiation therapy (SBRT) planning on a metastatic bone lesion.

Traditionally, systemic treatment is considered as the standard treatment for the majority of patients with metastatic cancer [6], including various treatment types ranging from chemotherapy to hormonal treatment and/or immunotherapy. In the past, diagnosis of metastatic disease ruled out any chance for cure, and patients would eventually succumb to their disease. More recently, it has been thought that cure might not be so unrealistic as first thought, at least for a subgroup of patients, as CheckMate 214 showed 10.5% complete responses with immune checkpoint inhibitors (ICI) in metastatic renal cell carcinoma (mRCC) patients, of which the majority ongoing after 30 months [7]. Furthermore, opposed to polymetastatic patients, oligometastatic patients have a more indolent disease course [2]. As a consequence, a different treatment strategy, called metastasis-directed therapy (MDT), has been developed. The rationale behind MDT is that the eradication of a low number of metastatic sites will hamper further metastatic dissemination, as it has been clearly demonstrated that metastases are an important source of further metastatic spread [8]. Reducing further metastatic spread by MDT might improve progression-free survival and overall survival [9]. Another advantage is the postponing of systemic treatment and, consequently, the accompanying side effects and financial burden, while keeping the patient’s quality-of-life unchanged. MDT can be achieved both by metastasectomy and stereotactic body radiotherapy (SBRT) [10, 11]. Compared to conventional radiotherapy, SBRT is delivered as a large dose in a few fractions, resulting in a high biological effective dose. Dosage is conformal around the target with a rapid fall-off doses away from the target to minimise the effect on the surrounding tissue [12]. For non-cranial metastases of the prostate and bladder, a total of three fractions of 10 Gy is prescribed, which can be reduced to three fractions of 9, 8 or 7 Gy if needed to respect the dose constraints to the surrounding organs of risk. For mRCC, a higher dosage of three times 14 Gy is attempted. An example of a radiation treatment planning can be found in Figure 1.

The use of MDT in treating oligometastatic RCC, bladder/urothelial cancer (UC), and prostate cancer (PCa) is gaining interest and popularity. In this topical review, we summarise the existing evidence of the treatment with MDT in these tumour types and give an overview of on-going trials that may change the treatment paradigm for these patients in the future.

## Renal cancer

RCC represents 3%–5% of adult cancers and accounts for more than 330,000 cases per year worldwide [13]. Up to 20% of patients have upfront metastatic disease and about 20%–40% of non-metastatic RCC patients will eventually develop metastasis, typically located in the lung, liver, bones and brain [14, 15]. Standard management for mRCC is based on ICI and/or targeted therapy [16]. Close active surveillance with deferred systemic treatment can be proposed in case of asymptomatic patients with limited disease burden. Before the availability of tyrosine kinase inhibitors and ICI, the prognosis of mRCC patients was poor with a median survival time ranging from 6 to 12 months, and even lower in case of brain metastases [17, 18]. However, currently a median OS up to 33 months has been reported [7]. Patients developing metachronous mRCC perform better than those presenting with synchronous mRCC [19].

While in the era of treatment with cytokines cytoreductive nephrectomy (CN) provided an OS benefit in the selected patients [17, 20], the role of CN needs to be re-established in the current era of immunotherapy and/or targeted therapy [20]. Multiple retrospective studies [22, 23] pointed towards an OS benefit for CN; however, the randomised trial CARMENA [24] demonstrated non-inferior OS when sunitinib alone was compared to CN followed by sunitinib in patients presenting with intermediate or poor-risk disease. Good performance status and good/intermediate International Metastatic Renal Cell Carcinoma Database Consortium and the Memorial Sloan Kettering Cancer Center risk classification were predictive for OS benefit with CN, suggesting that this subgroup of patients might still benefit from upfront CN. A second randomised trial (SURTIME [25]) was closed early due to poor accrual. In this trial, however, a trend towards an OS benefit was observed with CN performed in the absence of progression after 3 months of sunitinib, compared to immediate CN followed by sunitinib therapy. Until now, there are no prospective data on the combination of CN and immunotherapy. In a systematic review, it was stated that 13-30% of patients receiving upfront CN do not proceed to systemic therapy due to rapid disease progression or complications. Therefore, they concluded that upfront CN should be reserved for mRCC patients with only limited metastatic burden amenable to surveillance or metastasectomy, for the use of palliation or in patients with a favourable response or stable disease after initial systemic treatment [20]. Stereotactic body radiotherapy (SBRT) might be an alternative to CN in non-operable mRCC patients [26–28] with further prospective studies ongoing [14, 29].

In oligometastatic RCC, the distinction between synchronous and metachronous metastases is important. In case of synchronous oligometastatic disease, a multimodality treatment using a combination of CN, metastasectomy and systemic therapy might improve outcome in well-selected patients [30]. The prognosis of patients presenting with metachronous oligometastatic disease is better than those presenting with synchronous metastases, with time to development of the oligometastasis being an important prognostic factor [31–33]. Multiple retrospective studies demonstrated an improved survival using MDT for metachronous oligometastatic progression [34]. This knowledge, however, is not new. Two decades ago, Kavolius *et al* [35] reported long-term disease-free survival rates when metastasectomy was performed in patients presenting with oligometastatic RCC. The strongest predictors for prolonged survival are interval from nephrectomy to detection of metastasis, a single site of first recurrence, a solitary site of first metastasis, curative resection of first metastasis, and a metachronous presentation with recurrence [33, 35]. The surgery has been performed for metastases in different organs, such as lung, bone (isolated metastasis), brain, liver, adrenal gland, thyroid and pancreas [36–47]. After receiving a metastasectomy with complete resection, the 5-year survival benefit is 45% [16].

Besides metastasectomy, radiotherapy can be used for treating oligometastatic RCC [15]. Initially, radiation was expected to be less efficient as RCC metastasis are known to be radio-resistant. As much as this is the case for conventional radiotherapy, it holds no truth when considering SBRT. SBRT delivers a very high biological dose in a low number of fractions, resulting in a high dose per fraction. As a consequence, the cell membrane is the target (and no longer the cancer cell DNA itself). The stimulation of the ceramide pathway eventually leads to endothelial damage and tumour cell kill [48]. As an extra advantage, SBRT is able to stimulate the immune system [34, 49]. Different retrospective trials showed a good local control (90%–98%) with low toxicity. These studies emphasised the power of SBRT to postpone the need for systemic treatment [15, 50–56], with comparable results to surgery [57]. Both intracranial radiosurgery and extracranial SBRT appeared to be very well tolerated. Fit patients with extracranial metastasis treated after an initial period of deferring systemic treatment had the best prognosis [57]. There are only few prospective trials available [58]. A dose response relationship using SBRT has been suggested, with improved local control rates with delivery of a higher biologically effective dose[59].

There is currently no evidence to combine MDT with systemic treatment. Close surveillance is the recommended strategy after performing MDT. Trials investigating the combination of both SBRT and metastasectomy and checkpoint inhibitor immunotherapy are currently recruiting patients (Keynote 564, NCT01896271, NCT03065179, NCT02781506, NCT02855203, NCT03050060, NCT02318771, NCT02599779, NCT03149159, NCT02864615, NCT03469713 and NCT03226236). As RCC is considered an immunogenic tumour, the use of SBRT in the oligometastatic setting and the combination with immune therapy is particularly relevant. SBRT is considered to have an immunostimulating effect by converting immunologically cold tumours into hot tumours due to the combination of different mechanisms that increase tumour immunogenicity, overcome an immunosuppressive tumour microenvironment and recruit antigen-presenting and immune-effector cells to the tumour microenvironment [34]. This immunostimulating effect can also translate in the so-called abscopal effect, defined as the presence of an anti-tumour effect both inside and outside irradiation fields [34, 49, 60]. In the future, the use of biomarkers will be increasingly more important, as they can identify biological factors that may further improve patient selection.

Further prospective trials are needed to establish the role of SBRT or metastasectomy in metastatic RCC and to optimise the sequencing and combination of all treatment options in the era of targeted agents and immunomodulatory agents, and to identify the patients that will benefit the most from this approach.

## Urothelial bladder cancer

Bladder cancer accounts for over 430,000 new cases worldwide every year [61, 62], with urothelial carcinoma (UC) being the predominant histological subtype in more than 90% of the diagnoses [61]). About 25% of the bladder cancers present with muscle-invasive disease [63] with a high ability to metastasise [64]. Approximately 5% of patients presents with upfront metastatic disease [65]. The prognosis of metastatic bladder cancer is poor, with a 5-year overall survival (OS) rate of only 15%.

Despite the bad prognosis of metastatic UC (mUC), a subgroup of patients lives longer than 5 years after diagnosis. This subgroup consists of oligometastatic patients presenting with ≤3 metastatic lesions in a single organ (but no liver), and with the largest diameter of metastatic foci of ≤ 5 cm [66]. The critical question remains the definition of the imaging tool to optimise the finding of oligometastatic disease. Although fluorodeoxyglucose-positron emission tomography (PET)-computed tomography (CT) shows promising specificity and sensitivity, only small trials exist, and therefore it use remains controversial [64]).

MDT for mUC has been described in single-institution, small retrospective case series or observational reports [64]. Mostly, metastasectomy or SBRT was performed after platinum-based chemotherapy with only few reports on upfront metastasectomy [67]. A systematic review and meta-analysis reporting on 15 retrospective and 2 prospective trials, reported on metastasectomy in mUC [68]. Six of the studies included metastasectomy for different metastatic localisations, three studies reported on distant lymphadenectomy, five involved pulmonary metastasectomy and three reported on the resection of intracranial lesions. Lung metastases were the most common site of metastasectomy. Mean time to clinical relapse was 14 months and the overall survival from the resection of metastases reported in the different articles ranged from 2 to 60 months. Best results were observed in patients with a good response after chemotherapy (at least stable disease), in patients presenting with limited or solitary sites of metastases, and in patients presenting with the lymph node or lung metastases [68].

Shah *et al* [69] reported on the results of consolidating radiation therapy delivered to patients presenting with mUC after they received chemotherapy [69]). Of note, patient selection was highly selective as only 2 out of 22 patients had M1 disease (1 lung metastasis and 1 mediastinal lymphadenopathy). The remaining patients had N1–N3 disease. Median OS was 49 months with 36% of patients being disease-free after 6 years [69]). Manig *et al* [70] reported on the prognostic factors for survival in 63 patients with irradiated mUC [70]. Median overall survival after irradiation was 6 months. A radiation dose (<=20 Gy equivalent dose in 2 Gy fractions or EQD2) was the only significant factor in multivariate analysis negatively influencing overall survival. In another retrospective cohort by Augugliaro, 13 patients with 21 lesions were treated with SBRT or three-dimensional conformal radiation therapy (3D-CRT) for recurrent oligometastatic transitional cell carcinoma with lymph node, bone and lung lesions or local recurrence [71]. Radiological progression of disease was registered in nine patients at the median of 4.2 months (range 1.9–18.8). Leonetti *et al* [72] retrospectively identified seven patients who were treated with SBRT for nodal metastasis (14 lesions) with the aim of either to consolidate the response achieved by a previous systemic therapy or to delay the start of systemic chemotherapy. Five patients had bladder UC, two patients had upper tract UC. The treatment was atoxic. They reported a progression-free survival (PFS) of 2.9 months (95% CI 2.6–3.1) and median lesion progression-free survival, defined as local progression of the treated lesion, independently from the onset of new metastatic lesions, of 11.4 months (95% CI 3.4–19.4).

Based on these clinical results, guidelines have incorporated MDT as a treatment option in a highly selected group of oligometastatic patients [65].

Additionally, the combination of SBRT and ICI holds promise. A synergistic effect of this combination has been demonstrated in preclinical trials [73]. However, from a clinical viewpoint, we have to await results on toxicity and efficacy. A phase 1 trial with nine patients demonstrated that the combination of SBRT and pembrolizumab was safe (grade 3 toxicity in 1 patient, no dose limiting events). When SBRT was administered before the 3th cycle of pembrolizumab, the response of non-irradiated lesions was clearly better than when SBRT was administered before the start of pembrolizumab. From this study, one can assume that the abscopal effect is more likely to happen when SBRT and pembrolizumab are delivered concomitantly [74]). In this setting, several trials are ongoing. A clinical registry (NCT02170181), 5,000 patients will be enrolled who will receive SBRT with the aim to define patterns of care. Another trial is looking at the feasibility of pembrolizumab and SBRT in patients with advanced, platinum-refractory urothelial carcinoma (NCT03287050). CHEERS (NCT03511391) is a randomised controlled phase II trial investigating whether the addition of SBRT to ICI can improve progression-free survival as compared to ICI monotherapy. The combination of radiation therapy and ICI is currently also being investigated in the setting of locally advanced urothelial carcinoma of the bladder (RACE IT trial, NCT03529890). Another phase 2 trial is evaluating the use of radiation therapy and durvalumab with or without tremelimumab for the patients with bladder cancer that cannot be removed by surgery, has spread to nearby tissue or lymph nodes, or metastatic bladder cancer (NCT03601455).

## Prostate cancer

PCa is known to be a very heterogeneous disease, ranging from indolent and localised disease requiring only active surveillance or local treatment, to an aggressive type with high metastatic potential requiring multimodality treatment. Although approximately 80-85% of all prostate cancers are detected in a curable state, PCa still remains one of the leading causes of cancer-related mortality worldwide due to its high incidence [11]. Approximately 17% of patients with non-metastatic high-risk disease will develop metastases within 10 years after diagnosis [75] and about 4% of all new prostate cancer diagnoses contain upfront metastatic disease [76]. PCa can spread lymphogeneous and hematogenous with bone, lung and liver being the most frequent sites of distant metastases [77]. The presence of visceral metastatic lesions worsens prognosis [78]. The standard of care for metastatic hormone-sensitive prostate cancer consists of androgen-deprivation therapy (ADT) [79], whether or not combined with second-line hormonal treatment or chemotherapy in de-novo metastasised disease. Although a response is observed in 80%–90%, the majority of patients progresses to castration-refractory prostate cancer (CRPC) within 2–3 years [80, 81]. The median survival of these patients runs up to 35 months [82–84].

New insights have led to an improved understanding of the underlying mechanism of prostate carcinogenesis and metastasis and the contribution of tumour microenvironment in the metastatic process [85]. The increasing availability of imaging modalities as choline PET-CT and especially prostate-specific membrane antigen (PSMA) PET-CT empower the earlier detection of a single or a limited number of metastases [86] and consequently increase the incidence of oligometastatic prostate cancer. There exist more than 35 retrospective case series and single-institution reports [2, 87] reporting on oligometastatic prostate cancer and treatment with MDT, strengthened by several prospective randomised controlled trials [88–90].

Within oligometastatic prostate cancer, we define three different subsections: upfront oligometastatic disease, oligorecurrent prostate cancer and oligoprogression in CRPC.

### Upfront oligometastatic disease (hormone sensitive)

In de novo metastatic disease, ADT remains the cornerstone treatment [91]. Recent randomised trials demonstrated improved overall survival when chemotherapy or second-line androgen receptor target agents were added to ADT.

In case of low-volume metastatic disease, 3-year OS significantly increased from 73% to 81% when radiotherapy to the prostate (and consequently primary tumour) was added to ADT [92]. This benefit was also suggested in the HORRAD trial [93] which demonstrated, although not significant, an OS benefit with a hazard ratio similar to the STAMPEDE trial (HR 0.68, 95% CI 0.42 -1.10 and hazard ratio 0.68; 95% CI 0.52-0.90 respectively). The lack of significance is probably due to the fact that the trial was underpowered to be able to demonstrate a difference in the low-volume subgroup. Pooled data of HORRAD and STAMPEDE showed a 7% improvement in 3-year survival in men with fewer than five bone metastases [94]. Concerning radical prostatectomy as the treatment of the primary in this setting, only retrospective data exist [95–100]. However multiple trials are ongoing (NCT01957436 PEACE 1, NCT03988686, NCT01751438, NCT02454543, g-RAMPP and ISRCTN15704862 trombone). There are currently no data available on the combination of the treatment of the primary with abiraterone or enzalutamide, and only 18% of patients in the STAMPEDE trial received additional docetaxel. The PEACE-1 trial will address this issue (NCT01957436).

Few data exist on radical treatment for upfront oligometastatic PCa. In a small single-institution series, patients with upfront oligometastatic disease were treated with radiotherapy to the primary and to all bone metastasis in 18 out of 22 patients. Twelve out of 22 patients showed a clinical failure after a median of 23.6 months (15.3–106.1) from the start of ADT, while three patients became CRPC with a median time to castration resistance of 31 months. Prostate RT in oligometastatic patients was considered to be safe and to be able to offer long-lasting control [101]. Another single-institutional study retrospectively evaluated newly diagnosed prostate cancer with oligometastatic disease treated with a combination of HDR brachytherapy, MDT and ADT (36 months). 18 patients had a median follow-up time of 62.5 months, of which 16 patients experienced CRPC and 5 patients died of prostate cancer during follow-up. The 5-yr CRPC-FS and CSS was 64.4% and 87.9%, respectively [102].

A prospective phase 2 trial (NCT03298087) is currently investigating the role of MDT in patients with newly-diagnosed M1a/b prostate cancer, with 1–5 lesions (excluding pelvic lymph nodes) based on PSMA PET-CT imaging. Patients will receive local treatment with radical prostatectomy, limited duration systemic therapy for a total of six months (leuprolide, abiraterone acetate with prednisone and apalutamide), metastasis-directed SBRT and post-operative fractionated radiotherapy if one of the following features is present on the prostatectomy specimen: pT≥3a, (p)N1, or positive margins [103]. Primary endpoint is the percentage of patients achieving a serum PSA of < 0.05 ng/mL, 6 months after recovery of serum testosterone ≥150 ng/dL. The study aims at including 28 patients, with expected study completion date mid-2023. In a trial by John Hopkins (NCT02716974), the safety (grades 3–5 toxicity evaluation) of oligometastatic-treatment with neo-adjuvant ADT and chemotherapy up to 6 months, followed by local tumour control with prostatectomy +/- adjuvant radiotherapy and consolidative SBRT to oligometastatic lesions, will be evaluated. The new arm of the STAMPEDE trial will investigate the role of the addition of MDT for oligometastatic lesions in addition to surgery or radiotherapy to the prostate and androgen deprivation therapy (STAMPEDE arm M).

### Oligorecurrence

Approximately 17% of patients with high-risk local disease will eventually develop metastases within 10 years after diagnosis [75]. Guidelines consider ADT to be the treatment of choice in symptomatic patients. However, these guidelines approve the deferral of ADT being in case of asymptomatic patients with a PSA <50 ng/dL and a PSA doubling time of more than 12 months [104]. Novel imaging techniques such as choline PET-CT and certainly PSMA PET-CT have led to an increased and earlier diagnosis of metastatic lesions. There is evidence that 75% of patients with recurrence after primary therapy will have ≤3 involved metastatic sites [87]. This situation is termed oligorecurrent PCa and is defined as the development of a limited number of metastatic lesions after primary treatment with radical prostatectomy and/or radiotherapy (RT). Multiple retrospective trials have been published that address the feasibility, low toxicity rates and promising outcome of MDT in those patients. It has been shown that about 50% of patients are progression-free at 1–3 years, when treated with MDT on the oligorecurrent lesions [105].

The prospective phase II trial STOMP [88] compared MDT with surveillance with ADT-free survival as primary endpoint. In total, 62 patients with three or less oligorecurrent choline PET-CT detected lesions and primary controlled tumour were included. For a median follow-up time of 3 years (IQR 2.3–3.7 years), MDT resulted in a 10 months ADT-free survival benefit compared to the surveillance arm. There was no grade >1 toxicity. These findings were confirmed by the POPSTAR trial [89] that evaluated the feasibility and tolerability of patients with oligorecurrent prostate cancer being treated with single-fraction SBRT, with 97% of the patients receiving the prescribed treatment. This treatment was well tolerated with one patient developing grade 3 toxicity. In another prospective single-centre study [106], 57 patients were treated with SBRT for up to 3 PSMA-PET-confirmed oligometastases, with LN-only and bone-only disease in 37 (65%) and 18 (31%) patients, respectively. For a median follow-up of 16 months, this approach resulted in a median biochemical disease-free survival of 11 months, with 32% of the patients being free of biochemical failure (BF) at 15 months. All patients with BF (*n* = 43) underwent a subsequent PSMA-PET scan, which revealed no in-field failures. Nodal metastases tended to relapse in distant nodes and bone metastasis to other bony sites, with only 8 out of 43 failures being solitary for which repeat SBRT could be given. Recurrences to pelvic lymph nodes were generally considered for whole-pelvis RT and ADT instead of repeat SBRT [106]. Bowden *et al* [90] extended the number of lesions up to 5, in a large single-arm prospective trial, including 199 patients receiving SBRT. For a median follow-up time of 35.1 months, the median treatment escalation free-survival was 27.1 months.

Another prospective phase 2 trial ORIOLE trial (NCT02680587) randomised between observation versus stereotactic ablative radiation (SABR) for patients with 1–3 metastatic lesions on conventional imaging with a PSAdt <15 months, with first results presented at ASTRO 2019. SABR significantly improved PFS with a hazard ratio of 0.3 (95% CI 0.1–0.8) and appeared to be safe and well tolerated, with no grade 3 or higher adverse events identified. In this trial, a blinded PSMA PET-CT was performed at baseline and day 180, and those patients who received SABRT had variable coverage of occult PSMA radiotracer-avid lesions. Total consolidation of those lesions decreased the incidence of new metastases at 6 months [107].

Evidence for improvement in harder endpoints like OS was shown in the phase 2 trial SABR-COMET trial [9]. This randomised basket-trial included patients of different primary tumour origins, of which 16 (27%) Pca, and showed a significant effect on both PFS and OS outcome when oligometastatic lesions (defined as 1–5 metastatic lesions and controlled primary, *n* = 14) were treated with MDT, compared to palliative standard of care alone.

### Oligoprogression in castration-refractory prostate cancer

In patients treated with ADT, the sensitivity to castration will eventually disappear due to the out-selection of castration-resistant clones, leading to the CRPC state of disease. CRPC is defined as the biochemical and/or radiological progression while castrate serum testosterone is <50 ng/dL or 1.7 nmol/L [108]. The indefinite continuation of ADT in the CRPC setting is generally accepted, as two trials showed a survival benefit when ADT was sustained [109, 110]. Moreover, the reimbursement of all subsequent treatments depends on the testosterone castrate level.

State-of-the art treatment consists of docetaxel [111–113], ARTA both in pre- [114, 115] and in post-docetaxel [116, 117] setting, cabazitaxel [118] or radium-223 [119], while ADT is continued. Trials with treatment with 177-luthetium are ongoing, with very promising first results [120, 121].

Nevertheless, CRPC is considered to be a very heterogeneous disease, with many details on tumour cell biology yet to be discovered. A subgroup of patients presenting with oligoprogressive disease defined as the progression of a limited number of lesions while other metastatic lesions are controlled by the current systemic treatment, has been described, [1–3, 122]. These progressive lesions are thought to contain clonogens resistant to the ongoing systemic treatment. Therefore, an approach with MDT to these lesions (also referred to as PDT: progression-directed therapy to include progression of the primary tumour and/or local recurrence) might keep patients responsive to their ongoing systemic treatment. Consequently, the need for next-systemic treatment (NEST) might be delayed, thereby also delaying at least its potential side effects and financial costs. There are currently very few prospective data on this novel approach. However, published retrospective trials show a fairly impressive postponement of NEST. Our own research group reported on 30 patients being treated with PDT for oligoprogressive lesions, showing a PFS of 11 months, and a NEST-FS of 21 months [122]. Those data correspond to the findings of Triggiani *et al* [123]. Others have investigated oligoprogression both in CSPC and CRPC patients [124–130], with only limited information available on the CRPC patients. A case report demonstrated the potential of SBRT as an additional tool in long-term control of oligoprogressive disease in CRPC, while the systemic therapy could be preserved [131]. Tran *et al* [132] showed early prospective results with 30% 1-year NEST-FS in 17 CRPC patients. Yoshida reported on 23 patients receiving PDT for oligometastatic CRPC (1–3 lesions defined on whole-body diffusion-weighted magnetic resonance imaging, showing a PSA-response in 16 of 18 patients with intrapelvic recurrence and none in five patients with recurrence outside the pelvis. They conclude that PDT can be an effective treatment option and site localisation is an important factor [133]. A multicentre study investigated the role of MDT in 86 patients presenting with 1–5 oligoprogressive lesions (117 in total) [134]. Median distant progression-free survival after SBRT was 12.3 months (95% CI 5.5–19.1 months). One- and two-year distant progression-free survival was 52.3% and 33.7%, respectively. There was a median NEST-FS of 21.8 months (95% CI 17.8–25.8 months). In the University Hospitals of Leuven, a phase 2 trial will open soon (MEDCARE). Other prospective trials are ongoing to investigate the role of SBRT for oligometastatic CRPC (NCT02192788, NCT02759783, NCT02816983, NCT01859221 and NCT03644303) and others look specifically at the combination of SBRT and ARTA (NCT03449719 and NCT02685397)

## Conclusion

We reviewed the results of MDT of oligometastatic disease in renal, bladder and prostate cancer. It is a promising treatment, which can postpone systemic treatment for a substantial time without inducing toxicity. Where the costs of new systemic treatments reach several thousands of euros per month per patient with continuous use, the price of MDT will be around 4,000 euro for the total treatment in case of SBRT. Furthermore, as the treatment options are limited, next line treatment can be ‘saved’ for later on. It is important that the discussion for MDT is based on a multidisciplinary decision to select for the appropriate treatment, and physicians create time to discuss this approach and the rationale with their patients. New data are awaited and further investigations will clarify the potential of this more aggressive treatment approach. We look forward to the day that MDT for oligometastatic disease in urological cancer will, finally, become second-hand news.

## Conflicts of interest

The authors have no conflicts of interest.

## Funding

None.

## References

[1] Allesandro Conti, Carolina D’Elia, Monica Cheng (2017). Oligometastases in genitourinary tumors: recent insights and future molecular diagnostic approach. Eur Urol Suppl.

[2] Weichselbaum RR, Hellman S (2011). Oligometastases revisited. Nat Rev Clin Oncol.

[3] Singh D, Yi WS, Brasacchio RA (2004). Is there a favorable subset of patients with prostate cancer who develop oligometastases?. Int J Radiat Oncol Biol Phys.

[4] Schweizer MT, Zhou XC, Wang H (2013). Metastasis-free survival is associated with overall survival in men with PSA-recurrent prostate cancer treated with deferred androgen deprivation therapy. Ann Oncol.

[5] Niibe Y, Hayakawa K (2010). Oligometastases and oligo-recurrence: The new era of cancer therapy. Jpn J Clin Oncol.

[6] Cheung P (2016). Stereotactic body radiotherapy for oligoprogressive cancer. Br J Radiol.

[7] Motzer RJ, Tannir NM, McDermott DF (2018). Nivolumab plus Ipilimumab versus Sunitinib in advanced renal-cell carcinoma. N Engl J Med.

[8] Gundem G, Van Loo P, Kremeyer B (2105). The evolutionary history of lethal metastatic prostate cancer. Nature.

[9] Palma DA, Olson R, Harrow S (2019). Stereotactic ablative radiotherapy versus standard of care palliative treatment in patients with oligometastatic cancers (SABR-COMET): a randomised, phase 2, open-label trial. Lancet.

[10] Berkovic P, De Meerleer G, Delrue L (2013). Salvage stereotactic body radiotherapy for patients with limited prostate cancer metastases: deferring androgen deprivation therapy. Clin Genitourin Cancer.

[11] Malik NH, Keilty DM, Louie AV (2019). Stereotactic ablative radiotherapy versus metastasectomy for pulmonary metastases: guiding treatment in the oligometastatic era. J Thorac Dis.

[12] Benedict SH, Yenice KM, Followill D Stereotactic body radiation therapy: the report of AAPM Tast Group 101. Med Phys.

[13] Bray F, Ferlay J, Soerjomataram I (2018). Global cancer statistics 2018: GLOBOCAN estimates of incidence and mortality worldwide for 36 cancers in 185 countries. CA Cancer J Clin.

[14] Rühle A, Andratschke N, Siva S (2019). Is there a role for stereotactic radiotherapy in the treatment of renal cell carcinoma?. Clin Transl Radiat Oncol.

[15] Meyer E, Pasquier D, Bernadou G (2018). Stereotactic radiation therapy in the strategy of treatment of metastatic renal cell carcinoma: a study of the Getug group. Eur J Cancer.

[16] Loh J, Davis ID, Martin JM (2014). Extracranial oligometastatic renal cell carcinoma: Current management and future directions. Future Oncol.

[17] Flanigan RC, Salmon SE, Blumenstein BA (2001). Nephrectomy followed by interferon alfa-2b compared with interferon alfa-2b alone for metastatic renal-cell cancer. N Engl J Med.

[18] Decker DA, Decker V, Herskovic A (1984). Brain metastases in patients with renal cell carcinoma: prognosis and treatment. J Clin Oncol.

[19] Kim SH, Lee DE, Park B (2019). Survival of patients receiving systematic therapy for metachronous or synchronous metastatic renal cell carcinoma: A retrospective analysis. BMC Cancer.

[20] Mickisch GHJ, Garin A, Van Poppel H (2001). Radical nephrectomy plus interferon-alfa-based immunotherapy compared with interferon alfa alone in metastatic renal-cell carcinoma: a randomised trial. Lancet.

[21] Bhindi B, Abel EJ, Albiges L (2019). Systematic review of the role of cytoreductive nephrectomy in the targeted therapy era and beyond: an individualized approach to metastatic renal cell carcinoma. Eur Urol.

[22] Hanna N, Sun M, Meyer CP (2016). Survival analyses of patients with metastatic renal cancer treated with targeted therapy with or without cytoreductive nephrectomy: a national cancer data base study. J Clin Oncol.

[23] Heng DYC, Wells JC, Rini BI (2014). Cytoreductive nephrectomy in patients with synchronous metastases from renal cell carcinoma: Results from the International Metastatic Renal Cell Carcinoma Database Consortium. Eur Urol.

[24] Méjean A, Ravaud A, Thezenas S (2018). Sunitinib alone or after nephrectomy in metastatic renal-cell carcinoma. N Engl J Med.

[25] Bex A, Mulders P, Jewett M (2019). Comparison of immediate vs deferred cytoreductive nephrectomy in patients with synchronous metastatic renal cell carcinoma receiving sunitinib: the SURTIME Randomized Clinical Trial. JAMA Oncol.

[26] Correa RJM, Rodrigues GB, Chen H (2018). Stereotactic ablative radiotherapy (SABR) for large renal tumors. Am J Clin Oncol Cancer Clin Trials.

[27] Correa RJM, Ahmad B, Warner A (2018). A prospective phase I dose-escalation trial of stereotactic ablative radiotherapy (SABR) as an alternative to cytoreductive nephrectomy for inoperable patients with metastatic renal cell carcinoma. Radiat Oncol.

[28] Siva S, Pham D, Gill S (2012). A systematic review of stereotactic radiotherapy ablation for primary renal cell carcinoma. BJU Int.

[29] Francolini G, Detti B, Ingrosso G (2018). Stereotactic body radiation therapy (SBRT) on renal cell carcinoma, an overview of technical aspects, biological rationale and current literature. Crit Rev Oncol Hematol.

[30] Bex Axel, de Bruijn Roderick, Noe Allen (2017). Time to targeted therapy after cytoreductive nephrectomy (CN) and surveillance in patients with synchronous unresectable metastases of renal cell carcinoma (RCC). J Clin Oncol.

[31] Santoni M, Conti A, Porta C (2015). Sunitinib, pazopanib or sorafenib for the treatment of patients with late relapsing metastatic renal cell carcinoma. J Urol.

[32] Hofmann HS, Neef H, Krohe K (2005). Prognostic factors and survival after pulmonary resection of metastatic renal cell carcinoma. Eur Urol.

[33] Dabestani S, Marconi L, Hofmann F (2014). Local treatments for metastases of renal cell carcinoma: a systematic review. Lancet Oncol.

[34] Tselis N, Chatzikonstantinou G (2019). Treating the Chameleon: Radiotherapy in the management of Renal Cell Cancer. Clin Transl Radiat Oncol.

[35] Kavolius JP, Mastorakos DP, Pavlovich C (1998). Resection of metastatic renal cell carcinoma. J Clin Oncol.

[36] Alt AL, Boorjian SA, Lohse CM (2011). Survival after complete surgical resection of multiple metastases from renal cell carcinoma. Cancer.

[37] Yang JC, Abad J, Sherry R (2006). Treatment of oligometastases after successful immunotherapy. Semin Radiat Oncol.

[38] Karam JA, Rini BI, Varella L (2011). Metastasectomy after targeted therapy in patients with advanced renal cell carcinoma. J Urol.

[39] Russo AE, Untch BR, Kris MG (2019). Adrenal metastasectomy in the presence and absence of extraadrenal metastatic disease. Ann Surg.

[40] Kollender Y, Bickels J, Price WM (2000). Metastatic renal cell carcinoma of bone: indications and technique of surgical intervention. J Urol.

[41] Lin PP, Mirza AN, Lewis VO (2007). Patient survival after surgery for osseous metastases from renal cell carcinoma. J Bone Jt Surg - Ser A.

[42] Higuchi T, Yamamoto N, Hayashi K (2018). Long-term patient survival after the surgical treatment of bone and soft-tissue metastases from renal cell carcinoma. Bone Joint J.

[43] Vickers MM, Al-Harbi H, Choueiri TK (2013). Prognostic factors of survival for patients with metastatic renal cell carcinoma with brain metastases treated with targeted therapy: results from the international metastatic renal cell carcinoma database consortium. Clin Genitourin Cancer.

[44] Stief CG, Jáhne J, Hagemann JH (1997). Surgery for metachronous solitary liver metastases of renal cell carcinoma. J Urol.

[45] Alves A, Adam R, Majno P (2003). Hepatic resection for metastatic renal tumors: is it worthwhile?. Ann Surg Oncol.

[46] Tanis PJ, Van Der Gaag NA, Busch ORC (2009). Systematic review of pancreatic surgery for metastatic renal cell carcinoma. Br J Surg.

[47] Schrodter S, Hakenberg OW, Manseck A (2002). Outcome of surgical treatment of isolated local recurrence after radical nephrectomy for renal cell carcinoma. J Urol.

[48] De Meerleer G, Khoo V, Escudier B (2014). Radiotherapy for renal-cell carcinoma. Lancet Oncol.

[49] Formenti SC, Demaria S (2009). Systemic effects of local radiotherapy. Lancet Oncol.

[50] Kano H, Iyer A, Kondziolka D (2011). Outcome predictors of gamma knife radiosurgery for renal cell carcinoma metastases. Neurosurgery.

[51] Wersäll PJ, Blomgren H, Lax I (2005). Extracranial stereotactic radiotherapy for primary and metastatic renal cell carcinoma. Radiother Oncol.

[52] Ranck MC, Golden DW, Corbin KS (2013). Stereotactic body radiotherapy for the treatment of oligometastatic renal cell carcinoma. Am J Clin Oncol Cancer Clin Trials.

[53] Zelefsky MJ, Greco C, Motzer R (2012). Tumor control outcomes after hypofractionated and single-dose stereotactic image-guided intensity-modulated radiotherapy for extracranial metastases from renal cell carcinoma. Int J Radiat Oncol Biol Phys.

[54] Ikushima H, Tokuuye K, Sumi M (2000). Fractionated stereotactic radiotherapy of brain metastases from renal cell carcinoma. Int J Radiat Oncol Biol Phys.

[55] Cochran DC, Chan MD, Aklilu M (2012). The effect of targeted agents on outcomes in patients with brain metastases from renal cell carcinoma treated with Gamma Knife surgery: clinical article. J Neurosurg.

[56] Franzese C, Franceschini D, Di Brina L (2019). Role of stereotactic body radiation therapy for the management of oligometastatic renal cell carcinoma. J Urol.

[57] Stenman M, Sinclair G, Paavola P (2018). Overall survival after stereotactic radiotherapy or surgical metastasectomy in oligometastatic renal cell carcinoma patients treated at two Swedish centres 2005–2014. Radiother Oncol.

[58] Svedman C, Sandström P, Pisa P (2006). A prospective Phase II trial of using extracranial stereotactic radiotherapy in primary and metastatic renal cell carcinoma. Acta Oncol.

[59] Kothari G, Foroudi F, Gill S (2015). Outcomes of stereotactic radiotherapy for cranial and extracranial metastatic renal cell carcinoma: a systematic review. Acta Oncol.

[60] Schaue D, Ratikan JA, Iwamoto KS (2012). Maximizing tumor immunity with fractionated radiation. Int J Radiat Oncol Biol Phys.

[61] Siegl RL, Miller KD JA (2019). Cancer statistics. CA Cancer Clin J.

[62] Antoni S, Ferlay J, Soerjomataram I (2017). Bladder cancer incidence and mortality: a global overview and recent trends. Eur Urol.

[63] Smith AB, Deal AM, Woods ME (2014). Muscle-invasive bladder cancer: evaluating treatment and survival in the National Cancer Data Base. BJU Int.

[64] Mertens LS, Horenblas S (2017). Bladder cancer: oligometastases and imaging. Nat Rev Urol.

[65] Flaig TW, Spiess TW, Agarwal N (2020). Bladder Cancer, Version 3.2020, NCCN Clinical Practice Guidelines in Oncology. J Natl Compr Canc Netw.

[66] Ogihara K, Kikuchi E, Watanabe K (2017). Can urologists introduce the concept of “oligometastasis” for metastatic bladder cancer after total cystectomy?. Oncotarget.

[67] Decaestecker K, Fonteyne V, Oosterlinck W (2017). Perspective on cytoreduction and metastasis-directed therapy in node positive and metastatic urothelial carcinoma of the bladder. Transl Androl Urol.

[68] Patel V, Collazo Lorduy A, Stern A (2017). Survival after metastasectomy for metastatic urothelial carcinoma: a systematic review and meta-analysis. Bl Cancer.

[69] Shah S, Zhang CA, Hancock S (2017). Consolidative radiotherapy in metastatic urothelial cancer. Clin Genitourin Cancer.

[70] Manig L, Käsmann L, Janssen S (2016). Predicting survival after irradiation of metastases from transitional carcinoma of the bladder. Anticancer Res.

[71] Augugliaro M, Marvaso G, Ciardo D (2019). Recurrent oligometastatic transitional cell bladder carcinoma: is there room for radiotherapy?. Neoplasma.

[72] Leonetti A, D’Abbiero N, Baldari G (2018). Radiotherapy for the treatment of distant nodes metastases from oligometastatic urothelial cancer: a retrospective case series. Int J Urol.

[73] Herrera FG, Bourhis J, Coukos G (2017). Radiotherapy combination opportunities leveraging immunity for the next oncology practice. CA Cancer J Clin.

[74] Sundahl N, Vandekerkhove G, Decaestecker K (2019). Randomized phase 1 trial of pembrolizumab with sequential versus concomitant stereotactic body radiotherapy in metastatic urothelial carcinoma. Eur Urol.

[75] Cooperberg MR, Broering JM, Carroll PR (2009). Risk assessment for prostate cancer metastasis and mortality at the time of diagnosis. J Natl Cancer Inst.

[76] Buzzoni C, Auvinen A, Roobol MJ (2015). Metastatic prostate cancer incidence and prostate-specific antigen testing: new insights from the European Randomized Study of Screening for Prostate Cancer. Eur Urol.

[77] Bubendorf L, Schöpfer A, Wagner U (2000). Metastatic patterns of prostate cancer: An autopsy study of 1,589 patients. Hum Pathol.

[78] Shou J, Zhang Q, Wang S (2018). The prognosis of different distant metastases pattern in prostate cancer: a population based retrospective study. Prostate.

[79] Huggins C, Hodges CV (1941). Studies on prostatic cancer i. the effect of castration, of estrogen and of androgen injection on serum phosphatases in metastatic carcinoma of the prostate. Cancer Res.

[80] Harris WP, Mostaghel EA, Nelson PS (2009). Androgen deprivation therapy: Progress in understanding mechanisms of resistance and optimizing androgen depletion. Nat Clin Pract Urol.

[81] Chandrasekar T, Yang JC, Gao AC (2015). Mechanisms of resistance in castration-resistant prostate cancer (CRPC). Transl Androl Urol.

[82] Yamashita S, Kohjimoto Y, Iguchi T (2016). Prognostic factors and risk stratification in patients with castration-resistant prostate cancer receiving docetaxel-based chemotherapy. BMC Urol.

[83] Miyazawa Y, Sekine Y, Shimizu N (2019). An exploratory retrospective multicenter study of prognostic factors in mCRPC patients undergoing enzalutamide treatment: Focus on early PSA decline and kinetics at time of progression. Prostate.

[84] Halabi S, Lin C-Y, Kelly WK (2014). Updated prognostic model for predicting overall survival in first-line chemotherapy for patients with metastatic castration-resistant prostate cancer. J Clin Oncol.

[85] Santoni M, Piva F, Scarpelli M (2015). The origin of prostate metastases: emerging insights. Cancer Metastasis Rev.

[86] Ceci F, Herrmann K, Hadaschik B (2017). Therapy assessment in prostate cancer using choline and PSMA PET/CT. Eur J Nucl Med Mol Imaging.

[87] Foster CC, Weichselbaum RR, Pitroda SP (2019). Oligometastatic prostate cancer: reality or figment of imagination?. Cancer.

[88] Ost P, Reynders D, Decaestecker K (2018). Surveillance or metastasis-directed therapy for oligometastatic prostate cancer recurrence: a prospective, randomized, multicenter phase II trial. J Clin Oncol.

[89] Siva S, Bressel M, Murphy DG (2018). Stereotactic abative body radiotherapy (SABR) for oligometastatic prostate cancer: a prospective clinical trial. Eur Urol.

[90] Bowden P, See AW, Frydenberg M (2020). Fractionated stereotactic body radiotherapy for up to five prostate cancer oligometastases: interim outcomes of a prospective clinical trial. Int J Cancer.

[91] Pagliarulo V, Bracarda S, Eisenberger MA (2012). Contemporary role of androgen deprivation therapy for prostate cancer. Eur Urol.

[92] Parker CC, James ND, Brawley CD (2018). Radiotherapy to the primary tumour for newly diagnosed, metastatic prostate cancer (STAMPEDE): a randomised controlled phase 3 trial. Lancet.

[93] Boevé LMS, Hulshof MCCM, Vis AN (2019). Effect on survival of androgen deprivation therapy alone compared to androgen deprivation therapy combined with concurrent radiation therapy to the prostate in patients with primary bone metastatic prostate cancer in a Prospective Randomised Clinical Trial: data from the HORRAD Trial. Eur Urol.

[94] Burdett S, Boevé LM, Ingleby FC (2019). Prostate radiotherapy for metastatic hormone-sensitive prostate cancer: a STOPCAP systematic review and meta-analysis. Eur Urol.

[95] Sooriakumaran P, Karnes J, Stief C (2016). A multi-institutional analysis of perioperative outcomes in 106 men who underwent radical prostatectomy for distant metastatic prostate cancer at presentation. Eur Urol.

[96] Heidenreich A, Pfister D, Porres D (2015). Cytoreductive radical prostatectomy in patients with prostate cancer and low volume skeletal metastases: results of a feasibility and case-control study. J Urol.

[97] Gratzke C, Engel J, Stief CG (2014). Role of radical prostatectomy in metastatic prostate cancer: data from the munich cancer registry. Eur Urol.

[98] Culp SH, Schellhammer PF, Williams MB (2014). Might men diagnosed with metastatic prostate cancer benefit from definitive treatment of the primary tumor? A SEER-based study. Eur Urol.

[99] Fossati N, Trinh QD, Sammon J (2015). Identifying optimal candidates for local treatment of the primary tumor among patients diagnosed with metastatic prostate cancer: a SEER-based study. Eur Urol.

[100] Satkunasivam R, Kim AE, Desai M (2015). radical prostatectomy or external beam radiation therapy vs no local therapy for survival benefit in metastatic prostate cancer: a SEER-medicare analysis. J Urol.

[101] Riva G, Marvaso G, Augugliaro M (2017). Cytoreductive prostate radiotherapy in oligometastatic prostate cancer: a single centre analysis of toxicity and clinical outcome. Ecancermedicalscience.

[102] Tsumura H, Ishiyama H, Tabata K-I (2019). Long-term outcomes of combining prostate brachytherapy and metastasis-directed radiotherapy in newly diagnosed oligometastatic prostate cancer: a retrospective cohort study. Prostate.

[103] Parikh NR, Huiza C, Patel JS (2019). Systemic and tumor-directed therapy for oligometastatic prostate cancer: study protocol for a phase II trial for veterans with de novo oligometastatic disease. BMC Cancer.

[104] Mottet N, Cornford P, van den Bergh RCN EAU Guidelines: Prostate Cancer, Uroweb. https://uroweb.org/guideline/prostate-cancer/#6.

[105] Decaestecker K, De Meerleer G, Lambert B (2014). Repeated stereotactic body radiotherapy for oligometastatic prostate cancer recurrence. Radiat Oncol.

[106] Kneebone A, Hruby G, Ainsworth H (2018). stereotactic body radiotherapy for oligometastatic prostate cancer detected via prostate-specific membrane antigen positron emission tomography. Eur Urol Oncol.

[107] Philips R, Lim SJ, Shi WY (2019). Primary outcomes of a phase II randomized trial of observation versus stereotactic ablative radiation for oligometastatic prostate cancer (ORIOLE). Int J Radiat Oncol Biol Phys.

[108] Cornford P, Bellmunt J, Bolla M (2017). EAU-ESTRO-SIOG guidelines on prostate cancer. part ii: treatment of relapsing, metastatic, and castration-resistant prostate cancer. Eur Urol.

[109] Hussain M, Wolf M, Marshall E (1994). Effects of continued androgen-deprivation therapy and other prognostic factors on response and survival in phase II chemotherapy trials for hormone- refractory prostate cancer: a Southwest Oncology Group report. J Clin Oncol.

[110] Taylor CD, Elson P, Trump DL (1993). Importance of continued testicular suppression in hormone-refractory prostate cancer. J Clin Oncol.

[111] Tannock IF, de Wit R, Berry WR (2004). Docetaxel plus prednisone or mitoxantrone plus prednisone for advanced prostate cancer. N Engl J Med.

[112] Beer TM, Garzotto M, Henner WD (2004). Multiple cycles of intermittent chemotherapy in metastatic androgen-independent prostate cancer. Br J Cancer.

[113] Ohlmann CH, Ozgur E, Wille S (2006). Second-line chemotherapy with docetaxel for prostatespecific antigen (PSA) relapse in men with hormonerefractory prostate cancer (HRPC) previously treated with docetaxel-based chemotherapy. Eur Urol Suppl.

[114] Ryan CJ, Smith MR, Fizazi K (2015). Abiraterone acetate plus prednisone versus placebo plus prednisone in chemotherapy-naive men with metastatic castration-resistant prostate cancer (COU-AA-302): final overall survival analysis of a randomised, double-blind, placebo-controlled phase 3 study. Lancet Oncol.

[115] Froehner M, Wirth MP (2014). Enzalutamide in metastatic prostate cancer before chemotherapy. N Engl J Med.

[116] Scher HI, Fizazi K, Saad F (2012). Increased survival with enzalutamide in prostate cancer after chemotherapy. N Engl J Med.

[117] Fizazi K, Scher HI, Molina A (2012). Abiraterone acetate for treatment of metastatic castration-resistant prostate cancer: final overall survival analysis of the COU-AA-301 randomised, double-blind, placebo-controlled phase 3 study. Lancet Oncol.

[118] De Bono JS, Oudard S, Ozguroglu M (2010). Prednisone plus cabazitaxel or mitoxantrone for metastatic castration-resistant prostate cancer progressing after docetaxel treatment: a randomised open-label trial. Lancet.

[119] Parker C, Nilsson D, Heinrich S (2013). Alpha emitter radium-223 and survival in metastatic prostate cancer. N Engl J Med.

[120] Aghdam RA, Amoui M, Ghodsirad M (2019). Efficacy and safety of 177Lutetium-prostate-specific membrane antigen therapy in metastatic castration-resistant prostate cancer patients: First experience in West Asia—a prospective study. World J Nucl Med.

[121] Hofman MS, Violet J, Hicks RJ (2018). [ 177 Lu]-PSMA-617 radionuclide treatment in patients with metastatic castration-resistant prostate cancer (LuPSMA trial): a single-centre, single-arm, phase 2 study. Lancet Oncol.

[122] Berghen C, Joniau S, Ost P (2019). progression-directed therapy for oligoprogression in castration-refractory prostate cancer. Eur Urol Oncol.

[123] Triggiani L, Alongi F, Buglione M (2017). Efficacy of stereotactic body radiotherapy in oligorecurrent and in oligoprogressive prostate cancer: new evidence from a multicentric study. Br J Cancer.

[124] Muldermans JL, Romak LB, Kwon ED (2016). Stereotactic body radiation therapy for oligometastatic prostate cancer. Int J Radiat Oncol Biol Phys.

[125] Tabata KI, Niibe Y, Satoh T (2012). Radiotherapy for oligometastases and oligo-recurrence of bone in prostate cancer. Pulm Med.

[126] Ahmed KA, Barney BM, Davis BJ (2013). Stereotactic body radiation therapy in the treatment of oligometastatic prostate cancer. Front Oncol.

[127] Franzese C, Zucali PA, Di Brina L (2018). The efficacy of Stereotactic body radiation therapy and the impact of systemic treatments in oligometastatic patients from prostate cancer. Cancer Med.

[128] Kam TY, Chan OSH, Hung AWM (2019). Utilization of stereotactic ablative radiotherapy in oligometastatic & oligoprogressive skeletal metastases: results and pattern of failure. Asia Pac J Clin Oncol.

[129] Deek MP, Yu C, Phillips R (2019). Radiation therapy in the definitive management of oligometastatic prostate cancer: the Johns Hopkins experience. Int J Radiat Oncol Biol Phys.

[130] Moyer CL, Phillips R, Deek MP (2019). Stereotactic ablative radiation therapy for oligometastatic prostate cancer delays time-to-next systemic treatment. World J Urol.

[131] Nguyen T-C, Bajwa R, Bari S (2018). Stereotactic body radiation therapy for the treatment of oligoprogression on androgen receptor targeted therapy in castration-resistant prostate cancer. Oxford Med Case Rep.

[132] Tran PT, Leigh MC, Ryan P (2017). Stereotactic ablative radiation therapy for the treatment of oligometastatic prostate cancer. J Clin Oncol.

[133] Yoshida S, Takahara T, Arita Y (2019). Progressive site-directed therapy for castration-resistant prostate cancer: localization of the progressive site as a prognostic factor. Int J Radiat Oncol Biol Phys.

[134] Triggiani L, Mazzola R, Magrini SM (2019). Metastasis-directed stereotactic radiotherapy for oligoprogressive castration-resistant prostate cancer: a multicenter study. World J Urol.

